# Genome-wide association study of early-onset and late-onset postpartum depression: the IGEDEPP prospective study

**DOI:** 10.1192/j.eurpsy.2024.26

**Published:** 2024-04-01

**Authors:** Sarah Tebeka, Emilie Gloaguen, Jimmy Mullaert, Qin He, Anne Boland, Jean-Francois Deleuze, Camille Jamet, Nicolas Ramoz, Caroline Dubertret

**Affiliations:** 1 Université Paris Cité, Institute of Psychiatry and Neuroscience of Paris (IPNP), INSERM U1266, team 1, 75014 Paris, France; 2 Department of Psychiatry, AP-HP, Louis Mourier Hospital, Colombes, France; 3 Department of Epidemiology, Biostatistics and Clinical Research, AP-HP, Hôpital Bichat, Paris, France; 4 IAME, INSERM, Université Paris Cité, Paris, France; 5 CEA, Centre National de Recherche en Génomique Humaine (CNRGH), Université Paris-Saclay, Evry, France

**Keywords:** early-onset postpartum depression, late-onset postpartum depression, genome-wide association study, quantitative trait locus, Functional Mapping and Annotation of Genome-Wide Association Studies, polygenic risk score, genotype-tissue expression

## Abstract

Postpartum depression (PPD) appears at two peak periods: early-onset prior to 2 months after delivery and late-onset (2 months after delivery and beyond). The aim of our study is to evaluate the different genetic factors associated with early- and late-onset PPD. With the French multicenter interaction of gene and environment of depression during postpartum (IGEDEPP) cohort, we conducted a genome-wide association study (GWAS) on 234 women with early-onset PPD and 223 women with late-onset PPD, as well as 1,204 controls with no history of lifetime depression. We performed post-GWAS analyses: functional mapping and annotation of GWAS results using MAGMA thanks to Functional Mapping and Annotation of Genome-Wide Association Studies (FUMA), expression quantitative trait loci (QTL) analyses, mapping using data from the PsychENCODE and GTEx, and polygenic risk score (PRS) analysis based on published GWAS. We found two new significant candidate loci for early-onset PPD, rs6436132 in *PTPRN* gene on chromosome 2 and rs184644645 in *RAD18* on chromosome 14, respectively, and one region of interest with five significant associated SNPs in chromosome 20 for late-onset PPD. Variant rs6436132 is the most significant associated with early-onset PPD, and it is a QTL that significantly modifies the expression and splicing of the *PTPRN* gene in different brain tissues. We also found an enrichment of uterus tissue in the early expression of PPD genes. PRS analysis showed a genetic overlap between both early and late-onset PPD and major depressive disorder, but only early-onset PPD overlaps with bipolar disorder. Our study presents two GWAS separately, highlighting two candidate loci for early-onset PPD and one different region of interest for late-onset PPD. These results have important consequences in our understanding of these disorders, especially since our data reinforce the hormonal pathophysiological hypotheses for early-onset PPD.

## Introduction

Postpartum depression (PPD) is one of the main complications of perinatal period [1]. Two peaks of occurrence are described: early-onset PPD beginning in the first 2 months after childbirth and late-onset PPD beginning between the 2nd and 12th month postpartum [2–5]. Our study among the interaction of gene and environment of depression during postpartum (IGEDEPP) women, in the Paris areas (France), showed 1-year cumulative incidence of PPD of 18.1%, a prevalence of early-onset PPD of 8.3%, and a prevalence of late-onset PPD of 12.9% [6], in line with previous works [7]. Indeed, PPD is associated with significant morbidity and mortality for mothers: onset of chronic psychiatric disorders, non-psychiatric disorders, and mortality due to both natural and unnatural causes such as suicide [8]. Suicide is the cause of more than 4% of maternal deaths [9], and currently, the co-first cause of maternal death in France. Children of mothers with PPD may have eating or sleeping disorders and be at an increased risk for cognitive, psychomotor, emotional, and social development disorders [10–13]. While PPD is a major cause of maternal and pediatric morbidity needing prevention and treatment, its causes remain poorly known.

This disorder is a complex multifactorial disease with sociodemographic, environmental, psychiatric, and genetic determinants [1,14,15]. We have previously shown that sociodemographic, clinical, and environmental determinants of early- and late-onset PPD are different, suggesting partially overlapping pathophysiological mechanisms [15,16]. However, in both types of PPD, personal and family histories of depression are important risk factors for PPD, suggesting a genetic vulnerability. Family aggregation studies confirmed a 2 or 3-fold risk of PPD when a first-degree relative had a PPD history [17,18]. Twin studies estimated that PPD heritability is approximately 50%, a third of which is not shared with the genetic variance of depression in the general population; this result suggests that the genetic etiologies for perinatal depression and non-perinatal depression only partially overlap [19].

Numerous studies have focused on PPD candidate genes, in particular those encoding transporters, neurotransmitters or molecules involved in postpartum neurohormonal changes [20–23]. For instance, several studies have shown that functional polymorphisms of *SLC6A4/5-HTT* gene (*5HTTLPR* and *STin2-VNTR*) were associated with the risk of early-onset PPD but not with late-onset PPD [24–26]. However, PPD is a complex disease where several genes likely interact synergistically with a relatively minor effect exerted by each gene individually [27]. A pangenomic approach, without any a priori pathophysiological hypothesis, could confirm or disconfirm the involvement of genetic factors, and ultimately characterize unknown biological pathways.

Several genome-wide association studies (GWAS) have been carried out on depression in the general population, but none specifically in the postpartum population [28]. Recently, a meta-analysis carried out on 246,363 cases of major depressive disorder (MDD) and 561,190 controls identified 102 independent genetic variants and 15 sets of genes associated with depression, including genes and genetic pathways associated with synaptic structure and neurotransmission [29]. In an independent replication sample of 414,055 cases and 892,299 controls, 87 of the 102 associated variants were replicated [29]. A GWAS was published on perinatal depression, including depression during pregnancy and PPD up to 6 months postpartum, without identifying any associated genetic variants [30]. More recently, a meta-analysis of 18 European cohorts (including IGEDEPP data) failed to identify a genetic marker of PPD in the year following childbirth [31].

Classically, GWAS analysis identifies a set of variants, each contributing a small effect. In this context, the PRS allows all variants to be considered for further analysis in independent samples [32]. In addition, five studies have investigated the genetic overlap between perinatal psychiatric disorders and mood disorders, both MDD and bipolar disorder (BD) [30,33–36]. All of these studies found genetic overlap with MDD, although the specific phenotypes of perinatal psychiatric disorder used differed between studies. The results for BD were inconsistent: three studies found a genetic overlap of PPD with BD [33,35,30] and two studies did not [34,36]. These different conclusions may reflect the different clinical evaluations and/or perinatal phenotypes used. Indeed, different disorders are assessed in each of the studies: depression, mood disorders (including puerperal psychosis), and perinatal psychiatric disorders (considering the prescription of psychotropic drugs). The timeframe considered also differs among studies: some consider early postpartum only, others up to 1 year postpartum, and still others the entire peripartum period including pregnancy. Defining a precise and homogenous phenotype of PPD should help to better characterize its determinants and lead to a better understanding of its genetic architecture.

Our work is based on data from IGEDEPP, which is the largest prospective multicenter cohort of French postpartum women with follow-up for 1 year. Assessment for depression was performed at three time points (immediately after childbirth, 8 weeks, and 1 year postpartum) by a trained clinician using reliable DSM-5 criteria. We hypothesize that early-onset PPD and late-onset PPD are two phenotypes that have distinct genetic substrates. We reported here the results of the first GWAS on PPD distinguishing early and late-onset PPD using an extremely dense single nucleotide polymorphism (SNP) genotyping array. Finally, post-GWAS analysis was performed for significant associated regions by using Functional Mapping and Annotation of Genome-Wide Association Studies (FUMA) [37] and gene expression analyses to identify quantitative trait loci (QTL) using GTEx-portal and PsychENCODE.

## Patients and methods

### Patients

IGEDEPP is a French prospective cohort of 3,310 women from 8 maternity departments in the Paris metropolitan area and included between November 2011 and June 2016. All of the women were over 18 years old, French speaking, and of European ancestry (defined by self-declaration of the four grandparents born in Europe).

Women were evaluated at three-time points by a clinician trained to assess depression using the semi-structured interview of the Diagnostic Interview for Genetic Studies (DIGS), based on DSM-5 criteria [38]. Among the women included in the maternity department between the 2nd and 5th day postpartum, 91.1% were followed up at 8 weeks postpartum to assess early-onset PPD, and 71.0% at 1 year after childbirth to assess late-onset PPD. A blood sample was taken at the maternity department after informed consent was received. Further details regarding this cohort can be found in [6].

The research protocol (ClinicalTrials.gov, Identifier: NCT01648816), including informed consent procedures, was approved by the Data Protection and Freedom of Information Commissions and the French Ethics Committee (Ile de France I).

### Definition of cases and controls

In the present work, we included only women who had a clinical diagnosis of early and late-onset PPD (N = 1,661) to define cases and controls.

Among the cases, 234 met the criteria for early-onset PPD (presence of a major depressive episode with onset from delivery up to 2 months postpartum), and 223 met the criteria for late-onset PPD (presence of a major depressive episode with onset between 2 months and 1 year postpartum). Early- and late-onset PPD were exclusive categories.

The control group consisted of 1,204 women evaluated at the same three times points as the PPD group (at the maternity department, 8-week postpartum and at 1-year postpartum) and who had not presented a depressive episode within their lifetime: either before pregnancy, during pregnancy, or postpartum (early and late). These controls are considered “super controls” for this investigation because no depression was reported at any point during their lifetime.

### DNA extraction and genotyping

DNA was extracted from blood samples and genotyping was performed using the Illumina Global Screening Array with a multidisease drop-in panel (GSA-MD v1.0). Genotyping was performed at the CNRGH (Evry, France) on an Illumina automated high-throughput genotyping platform, according to manufacturer’s instructions. To validate results after imputation, two imputed SNPs (rs6028723 and rs6129447) on chromosome 20 were genotyped by TaqMan® SNP genotyping assays (Thermo Fisher Scientific) using an Applied Biosystems 7900HT Sequence detection system. Taqman genotyping was also performed at CNRGH.

### Quality control and imputation

Quality control (QC) was performed for all patients all genotyped patients, regardless of their known PPD status, according to the Turner et al recommendations [39]. Genotype imputation was performed using the pre-phasing/imputation stepwise approach implemented in IMPUTE2/SHAPEIT, and imputation reference set was 1000Genomes/phase 3 [40].

The number of SNPs and samples excluded at each stage of QC before and after imputation, as well as their parameters, are described in Figure S1 and in the Supplementary methods section. Of the 687,571 variants genotyped by the chip, 288,039 were selected after this QC (note that 388,277 variants were excluded because they had an MAF ≤ 0.05, Figure S2), and 50 patients were removed from subsequent analysis. More than 10 million markers were added through imputation, and 7.1 million were retained by QC performed after imputation.

A principal component analysis (PCA) was performed on a collection of SNPs (detailed in Supplementary methods and reported in Figure S3).

### Genome-wide association analyses

The association analysis was conducted with a general regression model (GRM), which corresponds to a corresponds to a 2-degree of freedom test based on a logistic regression model that allows for the testing of an association without assumption of the genetic model and avoids loss of power in the case of deviation from additivity. The GRM used an additive and a dominance deviation term for the logistic regression, with the coding of 0/1/2 for the additive term (Add) and 0/1/0 for the dominance deviation term (DomDev) for AA, Aa, and aa genotypes, respectively, with a risk allele. GRM allows to test the genetic model in a second time [41,42]: comparison of the regression coefficient of Add and DomDev terms in a case of association test allowed for the selection of the best genetic model. Despite testing multiple genetic models, the GRM procedure yields *p*-values for association that do not need to be adjusted for multiple tests. The statistical significance thresholds were 5 × 10^–8^ and 0.01 for SNP-phenotype association assessment and genetic model selection, respectively. All imputed SNPs that passed quality control were tested without post-imputation pruning. For each significant marker, the association was tested a second time with the selected genetic model to obtain odd ratios (ORs) and 95% confidence interval (CI) from asymptotic approximation. The percentage of explained variance was estimated based on the selected genetic models.

For the two imputed genotyped markers, the association was tested directly with the selected genetic model. For each phenotype, a further GWAS analysis was performed using the most significant SNP as a covariate.

A sensitivity analysis, where 8 individuals that seem to be outliers according to Figure S3 are removed, has also been conducted.

Quality controls and association analysis were carried out using Plink software (version 1.9) [43], and R console, Manhattan plot was performed by the qqman package [39], and regional plots and *R* ^2^ estimations were obtained using LDlink [44].

### Post-GWAS analyses

To evaluate the functional impact of candidate markers, FUMA (v1.3.7) [37] was used with default settings, with gene-based, gene-pathway, and tissue-enrichment analyses performed by MAGMA [45]. Correction for gene analysis was calculated by Bonferroni correction as 0.05/number of tested genes (with 17766 genes, the threshold was 2.8 × 10^–6^). Tissue-enrichment analysis included genotype-tissue expression (GTEx) v8 expression data [46] and BrainSpan [47].

We used other approaches to determine whether the genes identified in our study had been previously associated with psychiatric phenotypes: a literature search in PubMed (http://www.ncbi.nlm.nih.gov/pubmed/) and querying of the NCBI Gene database (http://www.ncbi.nlm.nih.gov/gene) to obtain information on the encoded proteins and their expression.

To identify the potential causal effect of candidate marker, the expression and splicing of quantitative trait locus (eQTL and sQTL, respectively) of significant SNPs were assessed by the GTEx v8 database (https://gtexportal.org/home/) and PsychENCODE (http://www.psychencode.org/). Significant markers rsIDs and genes of interest identified in the GWAS were submitted to the two selected databases. Description of the database is included in the Supplementary methods section.

### Polygenic risk score (PRS)

The PRSs of BD and MDD in both the early- and late-onset PPD cohorts were calculated using PRSice (v 2.3.3) [48] with the summary statistics from the published GWAS related on BD [49] and MDD [29] freely available online on the website from the Psychiatric Genomics Consortium (PGC; https://www.med.unc.edu/pgc/download-results/). PRS was calculated using 14 *p*-value thresholds (1, 0.5, 0.4, 0.3, 0.2, 0.1, 0.05, 0.01, 0.001, 10e−4, 10e−5, 10e−4, 10e−6, 10e−7, 10e−8) and we chose the best-fit threshold in general linear regression for each GWAS. Clumping was performed to remove the SNPs in linkage disequilibrium by including the 503 European samples from 1,000 genome projects as reference [50]. General linear regression models were used to determine the relationships between PPD status and each PRS, adjusted on age, and the top 10 principal components that indicate population structure were calculated using Plink. PRS were normalized using *R* to have a mean of 0 and a standard deviation of 1, in order to standardize the effect-size estimates. Empirical *p*-value was obtained through permutation of 10,000 times in PRSice. The statistical analyses were performed using *R* 4.0.5, and the plot was generated using the “Seaborn” module in Python 3.

### Ethics, consent, and permissions

The authors assert that all procedures contributing to this work comply with the ethical standards of the relevant national and institutional committees on human experimentation and with the Helsinki Declaration of 1975, as revised in 2008. All procedures involving human subjects were approved by the French Ethics Committee (Ile de France I) in March 2011 (reference numbers: AOM10056, NI10018, IGEDEPP, ID-RCB 2010-AO1315-34) and by Data Protection and Freedom of Information Commissions.

## Results

### Sample description

Among the 1,661 women who were included in our analyses, 234 (14.1%) had an early-onset PPD and 223 (13.4%) had a late-onset PPD. The controls were 1,204 (72.5%) without PPD.

The sociodemographic characteristics of the sample (N = 1,661) are described in Table 1. Participants had an average age of 32.7 years old, with 4.2% of women in our sample being less than 26, and 4.9% more than 39 years old. Women were mostly married or living common-law (97.3%), had a high level of education (93.4% reported at least a high school level), and most had a professional activity (93.9%).

**Table 1.** Sociodemographic characteristics among IGEDEPP cohort respondents included in GWAS (N = 1661)

	IGEDEPP GWAS N = 1,661	Controls N = 1,204	Early-onset PPD N = 234	Late-onset PPD N = 223
Age (y)				
25 or less	70 (4.2)	45 (3.7)	12 (5.1)	13 (5.8)
26–34	1,179 (71.0)	868 (72.1)	159 (67.9)	152 (68.2)
35–39	330 (19.9)	236 (19.6)	48 (20.5)	46 (20.6)
40 or more	82 (4.9)	55 (4.6)	15 (6.4)	12 (5.4)
Marital status				
Married, common-law married	1,616 (97.3)	1,176 (97.7)	222 (94.9)	218 (97.8)
Widowed, divorced, separated, never married	45 (2.7)	28 (2.3)	12 (5.1)	5 (2.2)
Education level				
University	1,551 (93.4)	1,131 (93.9)	212 (90.6)	208 (93.3)
Primary or high school	110 (6.6)	73 (6.1)	22 (9.4)	15 (6.7)
Employment				
Unemployed	102 (6.1)	59 (4.9)	18 (7.7)	25 (11.2)
Employed	1,559 (93.9)	1,145 (95.1)	216 (62.3)	198 (88.8)

### Main genetic association results

#### Early-onset PPD

**Figure 1.**
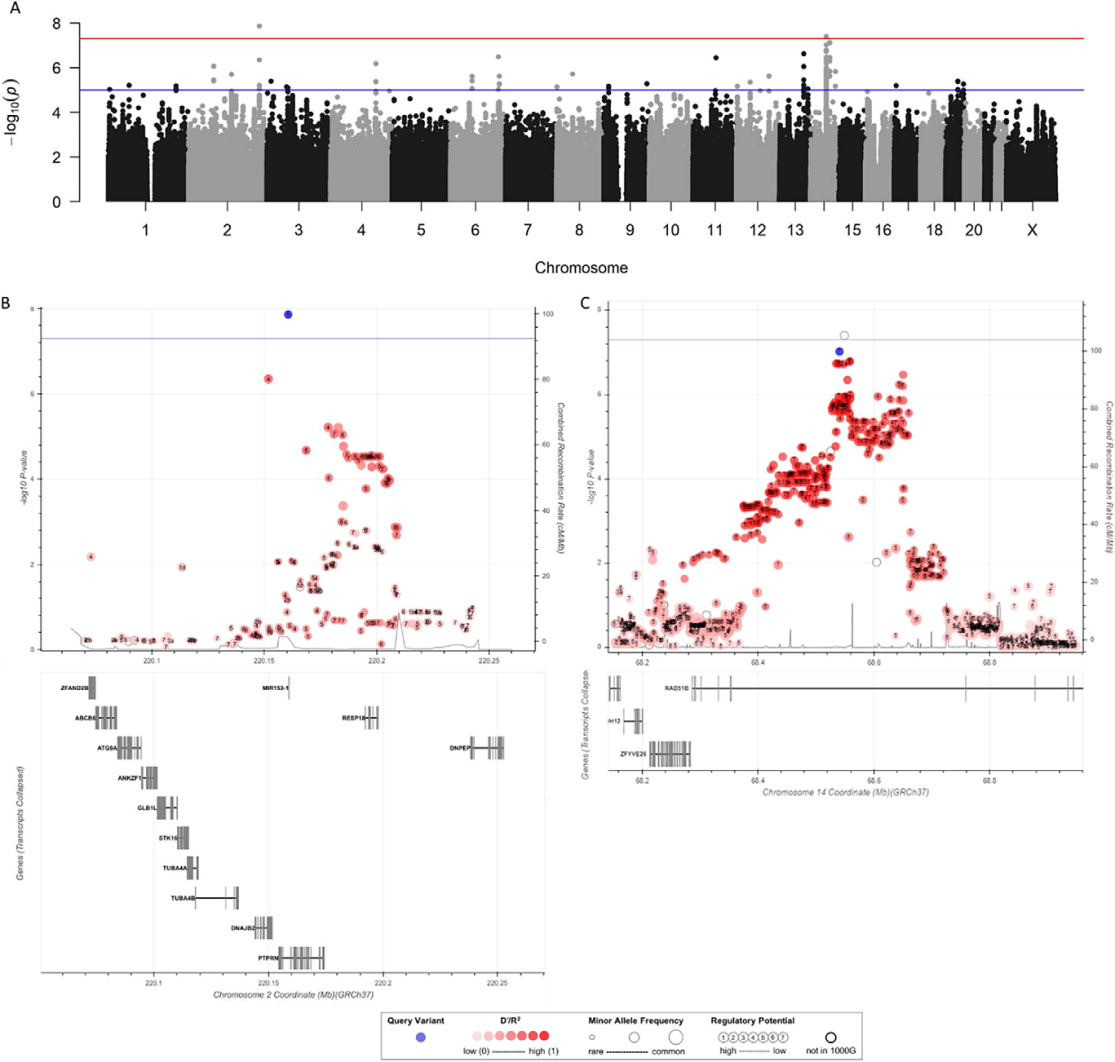
Associations with early-onset PPD. Manhattan plot of association with early-onset PPD (A). The red and blue lines corresponding to significant (5 × 10^−8^) and suggestive (1 × 10^−5^) threshold, respectively. Zoom plot for chromosome 2 (B) and for chromosome 14 (C). For Zoom plot, blue line corresponds to significant threshold (5 × 10^−8^). Best markers with data linkage disequilibrium (LD) data were indicated in blue, markers not present in 1000G database are presented in white. Variation of red correspond to the LD. Size of the point for each marker correspond to the minor allele frequency. Known genes are presented below the plot as line, indicating the location and size, with intronic and exonic part, of each gene.

We performed a GWAS on the early-onset PPD status (Figures 1 and S4(A)). Of note, the QQ-plots in Figure S4 show a slight inflation of the test statistics, which is not uncommon when using GRM, and is not observed when the analysis is carried out under a standard additive model (Figure S5).

The lead associated markers are presented in Table 2, section A. Using GRM, we found two significant markers associated with early-onset PPD, rs6436132 on chromosome 2 (2q35) (*P* = 1.3 × 10^–8^) and rs184644645 on chromosome 14 (14q24.1) (*p*-value = 3.9 × 10^–8^). The selected genetic model for these two markers was recessive. The sensitivity analysis, where 8 possible outliers are removed, gives similar results: the *p*-values of rs6436132 and rs184644645 were 4.7 × 10^–8^ and 2.3 × 10^–8^, respectively (Figure S6).

**Table 2.** Characteristics of the loci associated with the genotyping model and the best selected model (A) for early-onset PPD, (B) for late-onset PPD imputed and genotyped markers (B and C)

Chr	Lead SNP	Position^a^	Genotyping	Nearest gene^b^	Alleles^c^	Risk allele frequency		Beta Beta DomDev						
						Cases	Controls	Add	[CI 95%]	*P*-value DomDev	*P*-value GENO	Selected model	OR [CI 95%]	*P*-value best model
(A) Early–onset PPD														
2	rs6436132	20160644	Illumina	*PTPRN*; MIR153–1	**T**/G	0.33	0.26	0.57	−0.73 [−1.05 – −0.40]	1.2 × 10^−5^	1.3 × 10^−8^	Recessive	3.34 [2.20–5.07]	2.8 × 10^−9^
14	rs184644645	68549210	Imputed	*RAD51B*	**TTATG**/T	0.25	0.17	0.82	−0.64 [−1.04 – −0.24]	0.0016	3.9 × 10^−8^	Recessive	4.88 [2.70–8.82]	9.2 × 10^−9^
(B) Late–onset PPD														
20	rs6028719	38549047	Imputed	/	**T**/C	0.67	0.52	0.59	−0.095 [−0.42–0.23]	0.56	3.6 × 10^−8^	Additive	1.85 [1.49–2.30]	2.0 × 10^−8^
20	rs6028721	38550395	Imputed	/	**G**/C	0.67	0.52	0.59	−0.10 [−0.42–0.22]	0.54	3.6 × 10^−8^	Additive	1.85 [1.49–2.30]	2.1 × 10^−8^
20	rs6028723	38551618	Imputed	/	**C**/T	0.67	0.52	0.59	−0.089 [−0.41–0.23]	0.59	4.3 × 10^−8^	Additive	1.85 [1.49–2.30]	2.3 × 10^−8^
20	rs34294182	38553493	Imputed	/	**C**/–	0.67	0.52	0.59	−0.090 [−0.41–0.23]	0.58	3.9 × 10^−8^	Additive	1.85 [1.49–2.30]	2.1 × 10^−8^
20	rs6129447^d^	38566077	Imputed	/	**C**/A	0.67	0.52	0.58	−0.091 [−0.41–0.23]	0.58	8.3 × 10^−8^	Additive	1.83 [1.47–2.27]	4.3 × 10^−8^
(C) Late–onset PPD with genotyping SNP														
20	rs6028723	38551618	Genotyped	/	**C**/T	0.67	0.53	/	/	/	/	Additive	1.81 [1.46–2.41]	5.7 × 10^−8^
20	rs6129447	38566077	Genotyped	/	**C**/A	0.67	0.53	/	/	/	/	Additive	1.79 [1.44–2.22]	1.6 × 10^−7^

a Position on GRCH37.p13.

b The nearest genes around the variant according to dbSNP.

c Risk/protective allele in our sample.

d Non-significant marker with GRM, but selected for genotyping by CNG.

Under the recessive model, the rs6436132 marker has an OR of 3.34 (95% CI: [2.20–5.07]; *p*-value = 2.8 × 10^–9^), homozygous for TT, and was localized in an intronic variant of the *PTPRN* genes. Frequency of the T allele in our cohort was 0.33 for cases and 0.26 for controls. Two other genes (*RESP18* and *DNAJB2*) were present in the linkage disequilibrium region (*r* ^2^ > 0.6).

The rs184644645 marker has an OR of 4.88 (95% CI: [2.70–8.82]; *p*-value = 9.2 × 10^–9^) and homozygous for TATG insertion. It is an intronic variant of the *RAD51B* gene. Frequency of the TATG insertion in our cohort was 0.25 for cases and 0.17 for controls. No other gene was present in this region. A new genome-wide analysis using rs6436132 as a covariate did not yield a genome-wide significant marker (data not shown).

We note the effect size of these SNPs (consistent with our power study, Supplementary data), which explains 2.2% and 2.0% of the trait variance.

#### Late-onset PPD

**Figure 2.**
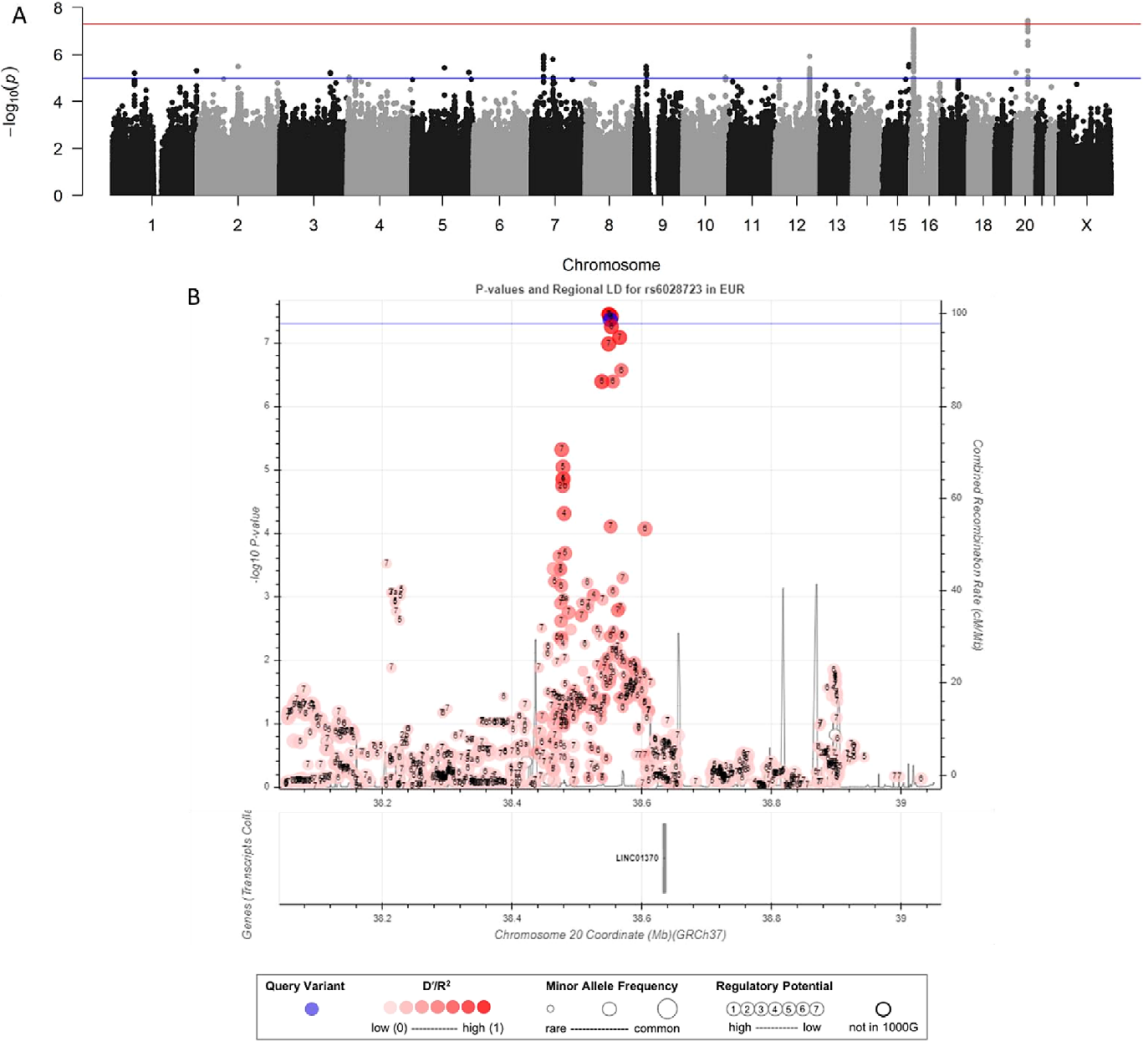
Associations with late-onset PPD. Manhattan plot of association with early-onset PPD (A). The red and blue line corresponding to significant (5 × 10^−8^) and suggestive (1 × 10^−5^) threshold, respectively. Zoom plot for chromosome 20 (B). For Zoom plot, blue line corresponds to significant threshold (5 × 10^−8^). Best markers with data linkage disequilibrium (LD) data were indicated in blue, markers not present in 1000G database are presented in white. Variation of red correspond to the LD. Size of the point for each marker correspond to the minor allele frequency. Known genes are presented below the plot as line, indicating the location and size, with intronic and exonic part, of each gene.

Results of the GWAS for late-onset PPD are displayed in Figures 2 and S4(B). We found five significant markers on chromosome 20 (20q12) presented in Table 2, section B. For these markers, the selected genetic model was additive. In the GWAS, these markers were imputed; to validate imputation, two of these significant markers in this region were genotyped. The results for imputed and genotyped data were consistent (Table 2, section C and Table S1). The two markers (rs6028723 and rsrs6129447) are significantly associated with late-onset PPD, respectively, with an OR of 1.81 (95% CI: [1.46–2.41]; *p*-value = 5.7 × 10^–8^) and 1.79 (95% CI: [1.44–2.22]; p-value = 1.6 × 10^–7^) for genotyped data. Only the long intergenic Non-Protein Coding RNA 1370 (LINC01370) is mapped in this region. A new genome-wide analysis using rs6028721 as a covariate yielded no genome-wide significant marker (data not shown). In the sensitivity analysis, where 8 possible outliers are removed, these SNPs do not reach statistical significance: the *p*-values range from 6 × 10^–8^ to 1.4 × 10^–7^ (Figure S7). The percentage of explained variance of this locus was estimated to be 2.2.

### Post-GWAS analysis

#### Early-onset PPD

**Figure 3.**
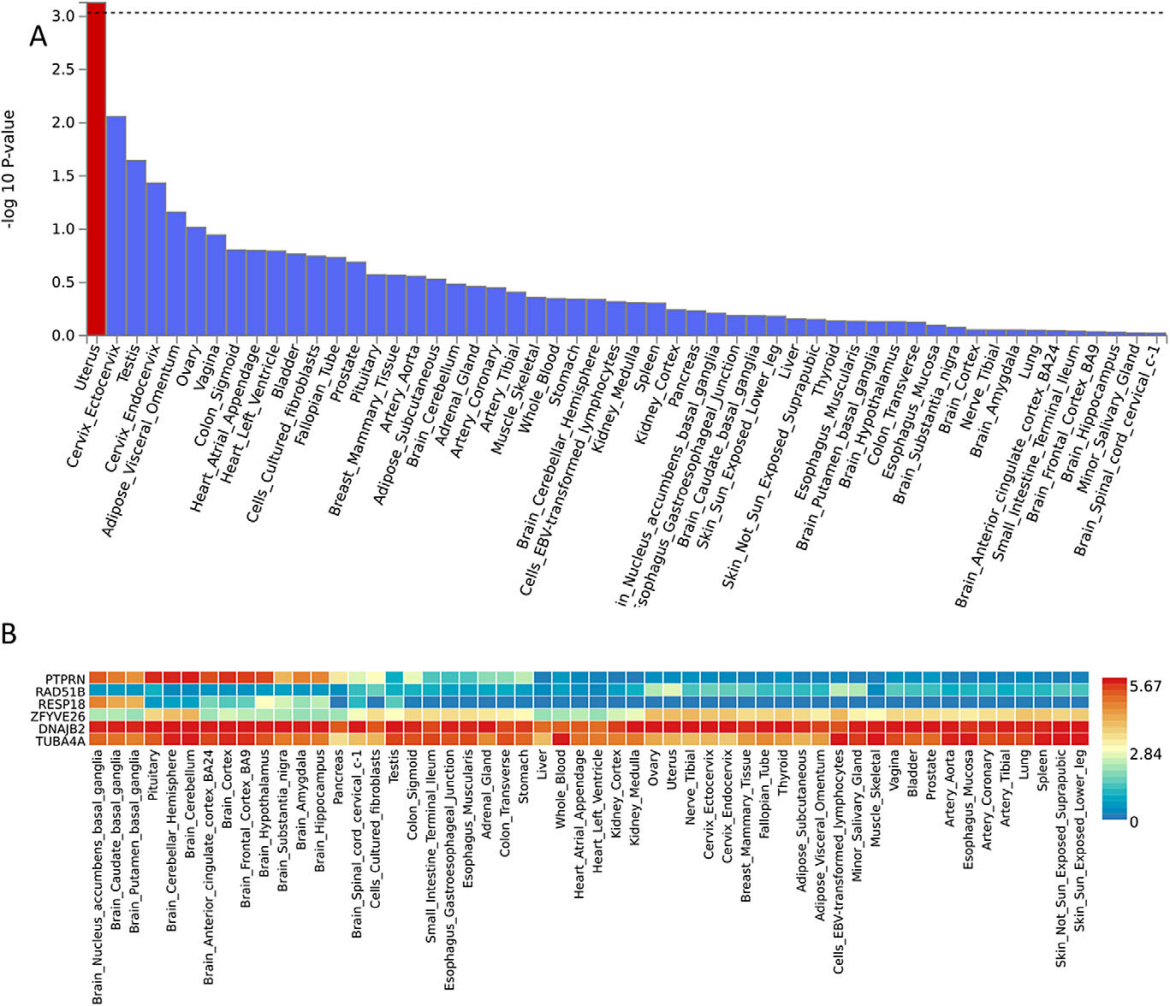
FUMA principal results for tissue expression. Tissue expression results on 53 specific tissue types by GTEx, with significantly enriched. Differentially expressed gene sets (*p*-value_corrected_ < 0.05) highlighted in red (FUMA) (A). Heatmap of expression of genes present in LD region associated with early-onset PPD (B). The expression level of the genes is between 0 (blue) and 5.67 (red).

The markers present on the GSA array mapped to 17,766 protein-coding genes. MAGMA Gene-Set analysis performed by FUMA did not identify any curated gene sets or GO terms significantly associated with early-onset PPD. In contrast, tissue expression analysis shows that genes differentially expressed in the uterus are significantly enriched with early-onset PPD (*p*-value = 7.4 × 10^–3^), but no brain tissue was identified (Figure 3A).

For the significantly associated regions, six genes were initially identified. Among these genes, the two genes present on chromosome 2 are preferentially and more strongly expressed in different brain cell tissues (*PTPRN* and *RESP18*) (Figure 3B). Genes located in chromosome 14 seem to be more ubiquitous.

Annotation of the chromatin performed by FUMA suggested no specific region of regulation as an enhancer for the 2q35 region, and chromatin state for the region 14q24.1 suggested the presence of enhancers and quiescent markers (Figure S8).

We also assessed whether genetic variants significantly associated with early-onset PPD exert their effects through the regulation of gene expression and splicing. Variant rs6436132 associated with early-onset PPD affects splicing and expression of *PTPRN*: sQTL and eQTL in the dorsolateral prefrontal cortex, *p* = 1.8 × 10^–25^ and 1.3 × 10^–9^, respectively, according to the PsyENCODE database (Table 3). Other nearby genes, including *RESP18* and *STK16*) were also found to be regulated by rs6436132, in several cerebral tissues (Table 3). Interestingly, using the PsychENCODE database, the search for sQTLs and eQTLs of the *PTPRN* gene identifies rs6436132 as the leading SNP with the most significant effect on expression among 120 significant results, and splicing among 28 significant results with a threshold 5 × 10^–8^ (data not shown).

**Table 3.** Significant QTL identified in PsychENCODE and GTEx database for the rs6436132 marker

PsychENCODE data						
Type of QTL	Gene	SNP	Chr	*p*		Tissue
sQTL	*PTPRN*	rs6436132	2	1.8 × 10^−25^		Dorsolateral Prefrontal Cortex
eQTL	*PTPRN*	rs6436132	2	1.3 × 10^−9^		Dorsolateral Prefrontal Cortex
GTEx data						
Type of QTL	Gene	SNP	Chr	*p*	NES	Tissue
sQTL	*PTPRN*	rs6436132	2	4.7 × 10^−9^	0.61	Brain–Cerebellum
eQTL	*PTPRN*	rs6436132	2	1.2 × 10^−12^	0.26	Brain–Nucleus accumbens
eQTL	*PTPRN*	rs6436132	2	2.1 × 10^−7^	0.19	Brain–Putamen
eQTL	*PTPRN*	rs6436132	2	4.1 × 10^−6^	0.16	Brain–Caudate
eQTL	*RESP18*	rs6436132	2	1.2 × 10^–14^	−0.80	Brain–Cortex
eQTL	*RESP18*	rs6436132	2	2.7 × 10^–13^	−0.78	Brain–Anterior cingulate cortex (BA24)
eQTL	*RESP18*	rs6436132	2	1.8 × 10^–11^	−0.54	Brain–Caudate
eQTL	*RESP18*	rs6436132	2	8.9 × 10^–11^	−0.70	Brain–Frontal Cortex (BA9)
eQTL	*RESP18*	rs6436132	2	2.8 × 10^–9^	−0.53	Brain–Nucleus accumbens
eQTL	*RESP18*	rs6436132	2	3.3 × 10^–9^	−0.53	Brain–Hypothalamus
eQTL	*RESP18*	rs6436132	2	8.4 × 10^–9^	−0.54	Brain–Putamen
eQTL	*RESP18*	rs6436132	2	1.2e–8	−0.35	Testis
eQTL	*RESP18*	rs6436132	2	5.3e–8	−0.69	Brain–Amygdala
eQTL	*RESP18*	rs6436132	2	2.7 × 10^–7^	−0.44	Brain–Hippocampus
eQTL	*STK16*	rs6436132	2	0.000043	−0.28	Brain–Putamen
eQTL	*GLB1L*	rs6436132	2	0.0000033	−0.24	Whole Blood

Chr, Chromosome; NES, normalized effect size (− underexpression, + overexpression).

No QTL was identified for the rs184644645 on chromosome 14.

#### Late-onset PPD

In contrast to the analysis of the early-onset PPD phenotype, MAGMA Gene-Set Analysis shows enrichment for genes associated with the regulation of cell adhesion molecule production (p-value corrected = 0.033) for late-onset PPD. However, the other analyses provided by FUMA do not show any significant results in terms of a difference in the level of expression both for the whole GWAS analyses and in the case of the precise analysis of the chromosome 20 region. The chromatin study, however, shows several markers associated with enhancers and a quiescent region of several genes (Figure S9).

None QTL was identified for the associated markers in chromosome 20.

### Polygenic risk score

PRS derived from both MDD GWAS and BD GWAS could discriminate early-onset PPD cases from controls, the variance explained by MDD had a larger effect size compared to that of BD (effect size = 0.32, empirical *p*-value <0.001 based on MDD GWAS and effect size = 0.22, empirical *p*-value = 0.033 based on BD GWAS). Only PRS from MDD GWAS could discriminate late-onset PPD cases from controls (effect size = 0.21, empirical *p*-value = 0.039) (Table S2 and Figure S10).

## Discussion

In this study, we performed two GWAS for early- and late-onset PPD separately. We distinguished these two phenotypes according to the timing of onset: 234 cases with early-onset PPD, 223 cases with late-onset PPD and 1,204 controls without a depression history. We highlighted two candidate loci for early-onset PPD and one region of interest for late-onset PPD. These SNP are not reported as significant in a recent meta-analysis on PPD [31], although the direction of effect is consistent with our findings. We also identified rs6436132 in chromosome 2 as a strong sQTL and eQTL potentially regulating the expression of *PTPRN* in the brain, and found an enrichment of uterus tissue in the early expression of PPD genes.

This first GWAS result reinforces the disputed idea that there is a distinction between these two phenotypes of PPD [51]. As it currently stands, some epidemiological, clinical and biological data support this point. Both GWAS on perinatal depression (including pregnancy and PPD until 6 month after delivery) and a meta-analysis of GWAS for PPD in the first year after delivery (thus mixing early- and late-onset PPD) failed to identify any specific genetic variants [30,31]. In addition, several previous candidate gene studies found different associations according to the phenotype: early or late-onset PPD [20–23]. For instance, several studies have been performed on the rs6265 polymorphism of *BDNF*, a gene encoding the brain-derived neurotrophic factor involved in cerebral neuroplasticity and the regulation of the hypothalamic–pituitary axis. An association between this polymorphism and PPD at 6–8 weeks was demonstrated, but was not found with late-onset PPD (evaluated at 3 and 6 months) [52–54]. Similarly, Tan et al. looked at the rs174575 polymorphism of the ESR2 gene, encoding the estrogen receptor, and found an association with early PPD at 6 weeks, but no association with late-onset PPD at 6 months [55]. This result may implicate hormonal changes in the immediate postpartum period in the pathophysiology of early-onset PPD [56].

Our GWAS reveals that early-onset PPD is significantly associated with two markers, on chromosome 2 (rs6436132) and chromosome 14 (rs184644645), respectively.

The rs6436132 marker lies in an intronic variant of the *PTPRN* gene, encoding the protein tyrosine phosphatase receptor-N, an autoantigen known to be signaling molecules that regulates a variety of cellular processes predominantly expressed in the brain, without a specific cerebral area. Interestingly, three other tyrosine phosphatase protein receptor genes (*PRPRR*, *PTPRD*, and *PTPRS)*, were found to be significantly associated with depressive disorder [57,58].

Eleven other genes in chromosome 2 were in the linkage disequilibrium in this region. The *RESP18* gene encodes for Regulated Endocrine Specific Protein 18, a protein sensitive to glucocorticoids in the corticotropic secretion pathway [59]. The “stress axis” is known to be involved in the pathophysiology of depression, in particular in PPD, with sudden changes in the hypothalamic–pituitary–adrenal and hormonal axes during early postpartum [60]. The expression of *RESP18* is mainly in the brain and particularly in the basal ganglia (nucleus accumbens, caudate, and putamen). Basal ganglia dysfunction was found in depression, particularly when psychomotor symptoms were present [61,62]. The putamen seems to be specifically involved in PPD [63,64]. Indeed, monamine oxidase A (MAO-A) total distribution volume was significantly elevated in the dorsal putamen during the early postpartum period. This may be a marker of a monoamine-lowering process that contributes to the change in mood [65]. Furthermore, *RESP18* is regulated by physiological factors such as blood sugar and dopaminergic drugs, but its functions remain unclear [66].

The second SNP associated with early-onset-PPD is rs184644645, an intronic variant of the *RAD51B* gene located on chromosome 14. It encodes a protein of the RAD51 family, conserved during evolution because it is essential for DNA repair. Its expression is ubiquitous. Polymorphisms of homologous recombination *RAD51* were found to be associated with cancer and rheumatoid arthritis [67,68], but never before in mood disorders.

The GWAS for late-onset PPD revealed five SNPs significantly associated with this phenotype in one region on chromosome 20. Two of them were confirmed by TaqMan genotyping. They were found in the Long Intergenic Non-Protein Coding RNA 1370 (LINC01370). No other genes or microRNAs were present in this region.

All of the risk variants reported for early-onset PPD or late-onset PPD found with our GWAS were located in non-exonic regions, suggesting that these genetic variations confer risk by modulating gene expression or splicing. Our analyses on QTLs showed that rs6436132 located on intron 18 of *PTPRN* is both: the SNP most associated with the early-onset PPD phenotype and a QTL that very significantly modifies the expression and splicing of the *PTPRN* gene in different brain tissues. This suggests that *PTPRN* might be a candidate causal gene in early-onset PPD.

Tissue expression analysis showed that genes differentially expressed in the uterus were significantly enriched and could confer risk specific to early-onset PPD. Interestingly, Kiewa et al. have demonstrated the potential role of genes expressed in the ovaries in perinatal depression [30]. Genes expressed in reproductive tissues, therefore, appear to have a specific implication in perinatal depression, particularly with the early-onset form, because this result is not found in MDD or late-onset PPD [29]. This further reinforces the hormonal pathophysiological hypotheses and a specific genetic vulnerability for early-onset PPD [56,69].

PRS analysis revealed a genetic overlap between PPD (both early and late-onset) and MDD. Although the question remains debated by some authors, this incomplete overlap gives more weight to the argument for a distinction between MDD and PPD [51,70]. We also found a genetic overlap with BD, though different than that with MDD. Interestingly, only early-onset PPD overlaps with BD; a finding which is consistent with our previous results that 12.2% of women with BD begin their disorder with PPD [71,72]. The differences between early and late-onset PPD displayed in the present study match those found in the literature. Byrne et al. and Pouget et al. also found a genetic overlap between early-onset PPD and BD [33,35]. These two studies focused on samples of women who developed symptoms early in postpartum: “around the time of delivery” and before the end of the first 3 months postpartum (including 80% before the first month postpartum), respectively. Conversely, Rantalainen et al. and Bauer et al., who considered women with later manifestations (within the first 6 months postpartum and within 1 year postpartum, respectively), did not show any genetic overlap between PPD and BD [34,36]. This may be explained by more rapid mood changes postpartum in women with BD [73].

Our study has several strengths. First, to our knowledge, this is the first GWAS to date on PPD, based on the largest multicenter prospective study of postpartum women. Second, our quality control showed a homogeneity of our cases and controls, suggesting that we can, with reasonable confidence, rule out a significant bias in our results caused by the residual genetic substructure. Third, the cases and controls were carefully selected: the diagnosis of PPD was made prospectively by a trained clinician and based on international DSM-5 criteria [74]. The date of depression onset was collected at 8 weeks for early-onset PPD, and at 1 year for late-onset PPD. Regarding the controls, in the IGEDEPP study, we favored “super controls”: women without any personal lifetime history of depression (evaluated using the DIGS while at the maternity hospital for the birth, then at 8 weeks, and 1 year postpartum, according to DSM-5 criteria). By using the most distinct phenotypes, we were able to highlight genetic differences and potentially explain previous discrepancies. Moreover, our population has particularly favorable sociodemographic characteristics: more than 90% of women were in a relationship, with a high level of education, and employed; these elements reduce the impact of a deleterious environment in revealing genetic vulnerabilities. Finally, our genetic analysis is based on the imputation of certain markers, the validity of this imputation was confirmed by genotyping, which reinforces the reliability of our data.

Our findings should be considered with certain limitations. First, our study was limited to women of European ancestry (self-reported) and is not representative of the French population as a whole, which limits the generalisability of our results to other populations. Second, our sample has a limited number of subjects for a GWAS. It has been shown that for multifactorial diseases, where several SNPs may be involved, each with a small effect, large sample sizes of the order of several thousand are needed to obtain sufficient statistical power [75,76]. Replication in an independent and larger sample is required and, for late-onset PPD, the significant results are not preserved in a sensitivity analysis. Finally, we do not have a functional analysis that could shed light on our results.

In summary, we report results from the first GWAS specifically for PPD. We found different markers for early- and late-onset PPD, identifying different markers that involve genes for each phenotype. We found two markers associated with early-onset PPD on chromosome 2 (*PTPRN* gene) and chromosome 14 (*RAD51B* gene), respectively, and five markers in one region on chromosome 20 associated with late-onset PPD. The distinction between early and late-onset PPD is reinforced by our PRS analyses: early-onset PPD has an extensive genetic overlap with both MDD and BD, while late-onset PPD only overlaps with MDD. Finally, we found an enrichment of uterus tissue in expression of early-onset PPD genes.

To conclude, these results provide an important step toward a better understanding of genetic factors of PPD and provide arguments for a distinction between early and late-onset phenotypes.

## Supporting information

Tebeka et al. supplementary material Tebeka et al. supplementary material

## Data Availability

The data that support the findings of this study are available from AP-HP. Restrictions apply to the availability of these data, which were used under license for this study. Data are available from the authors with the permission of AP-HP.

## Abbreviations

BD: bipolar disorder
CI: confidence interval
FUMA: Functional Mapping and Annotation of Genome-Wide Association Studies
GRM: general regression model
GTEx: genotype-tissue expression
GWAS: genome-wide association study
IGEDEPP: interaction of gene and environment of depression during postpartum
QTL: quantitative trait locus
OR: odd ratio
PPD: postpartum depression
PRS: polygenic risk score
SNP: single nucleotide polymorphism

## Supplementary material

The supplementary material for this article can be found at http://doi.org/10.1192/j.eurpsy.2024.26.

## References

[1] Howard LM, Molyneaux E, Dennis C-L, Rochat T, Stein A, Milgrom J. Non-psychotic mental disorders in the perinatal period. Lancet. 2014;384:1775–88. 25455248 10.1016/S0140-6736(14)61276-9

[2] Stowe ZN, Hostetter AL, Newport DJ. The onset of postpartum depression: Implications for clinical screening in obstetrical and primary care. Am J Obstet Gynecol. 2005;192:522–6. 15695997 10.1016/j.ajog.2004.07.054

[3] Kothari C, Wiley J, Moe A, Liepman MR, Tareen RS, Curtis A. Maternal depression is not just a problem early on. Public Health. 2016;137:154–61. 26972518 10.1016/j.puhe.2016.01.003

[4] Wikman A, Axfors C, Iliadis SI, Cox J, Fransson E, Skalkidou A. Characteristics of women with different perinatal depression trajectories. J Neurosci Res. 2019;98(7):1268–82. 30723972 10.1002/jnr.24390

[5] Rosenwald GC, Stonehill MW. Early and late postpartum illnesses. Psychosom Med. 1972;34:129–37. 5017101 10.1097/00006842-197203000-00006

[6] Tebeka S, Le Strat Y, De Premorel Higgons A, Benachi A, Dommergues M, Kayem G, et al. Prevalence and incidence of postpartum depression, and its environment factors: the IGEDEPP cohort. J Psychiatr Res. 2020;138:366–74. 10.1016/j.jpsychires.2021.04.004 33932643

[7] Norhayati MN, Hazlina NHN, Asrenee AR, Emilin WMAW. Magnitude and risk factors for postpartum symptoms: a literature review. J Affect Disord. 2015;175:34–52. 25590764 10.1016/j.jad.2014.12.041

[8] Vacheron M-N, Tessier V, Rossignol M, Deneux-Tharaux C. Comité National d’Experts sur la Mortalité Maternelle. [Maternal deaths due to suicide in France 2013–2015]. Gynecol Obstet Fertil Senol. 2021;49:38–46. 33161187 10.1016/j.gofs.2020.11.008

[9] Metz TD, Rovner P, Hoffman MC, Allshouse AA, Beckwith KM, Binswanger IA. Maternal deaths from suicide and overdose in Colorado, 2004–2012. Obstet Gynecol. 2016;128:1233–40. 27824771 10.1097/AOG.0000000000001695 PMC5121076

[10] Grace SL, Evindar A, Stewart DE. The effect of postpartum depression on child cognitive development and behavior: a review and critical analysis of the literature. Arch Womens Ment Health. 2003;6:263–74. 14628179 10.1007/s00737-003-0024-6

[11] Slomian J, Honvo G, Emonts P, Reginster J-Y, Bruyère O. Consequences of maternal postpartum depression: A systematic review of maternal and infant outcomes. Womens Health Lond Engl. 2019;15:1745506519844044. 31035856 10.1177/1745506519844044 PMC6492376

[12] Netsi E, Pearson RM, Murray L, Cooper P, Craske MG, Stein A. Association of persistent and severe postnatal depression with child outcomes. JAMA Psychiatry. 2018;75:247–53. 29387878 10.1001/jamapsychiatry.2017.4363 PMC5885957

[13] Stein A, Pearson RM, Goodman SH, Rapa E, Rahman A, McCallum M, et al. Effects of perinatal mental disorders on the fetus and child. Lancet. 2014;384:1800–19. 25455250 10.1016/S0140-6736(14)61277-0

[14] Tebeka S, Le Strat Y, Dubertret C. Developmental trajectories of pregnant and postpartum depression in an epidemiologic survey. J Affect Disord. 2016:62–8. 10.1016/j.jad.2016.05.058 27280964

[15] Tebeka S, Strat YL, Mandelbrot L, Benachi A, Dommergues M, Kayem G, et al. Early and late postpartum depression exhibit distinct correlates: the IGEDEPP prospective cohort study. BJOG Int J Obstet Gynaecol. 2021. doi:10.22541/au.159188592.22056384. 33656796

[16] Tebeka S, Le Strat Y, Etain B, Ray M, Mullaert J, Dubertret C, et al. Childhood trauma and perinatal depression: data from the IGEDEPP cohort. J Clin Psychiatry. 2021;82:20m13664. 10.4088/JCP.20m13664 34496464

[17] Murphy-Eberenz K, Zandi PP, March D, Crowe RR, Scheftner WA, Alexander M, et al. Is perinatal depression familial? J Affect Disord. 2006;90:49–55. 16337009 10.1016/j.jad.2005.10.006

[18] Payne JL, MacKinnon DF, Mondimore FM, McInnis MG, Schweizer B, Zamoiski RB, et al. Familial aggregation of postpartum mood symptoms in bipolar disorder pedigrees. Bipolar Disord. 2008;10:38–44. 18199240 10.1111/j.1399-5618.2008.00455.x PMC6999822

[19] Viktorin A, Meltzer-Brody S, Kuja-Halkola R, Sullivan PF, Landen M, Lichtenstein P, et al. Heritability of perinatal depression and genetic overlap with nonperinatal depression. Am J Psychiatry. 2016;173:158–65. 26337037 10.1176/appi.ajp.2015.15010085

[20] Elwood J, Murray E, Bell A, Sinclair M, Kernohan WG, Stockdale J. A systematic review investigating if genetic or epigenetic markers are associated with postnatal depression. J Affect Disord. 2019;253:51–62. 31029013 10.1016/j.jad.2019.04.059

[21] eCouto TC, Brancaglion MYM, Alvim-Soares A, Moreira L, Garcia FD, Nicolato R, et al. Postpartum depression: A systematic review of the genetics involved. World J Psychiatry. 2015;5:103–11. 25815259 10.5498/wjp.v5.i1.103 PMC4369539

[22] McEvoy K, Osborne LM, Nanavati J, Payne JL. Reproductive affective disorders: a review of the genetic evidence for premenstrual dysphoric disorder and postpartum depression. Curr Psychiatry Rep. 2017;19:94. 29082433 10.1007/s11920-017-0852-0

[23] Figueiredo FP, Parada AP, de Araujo LF, Silva Jr WA, Del-Ben CM. The Influence of genetic factors on peripartum depression: A systematic review. J Affect Disord. 2015;172:265–73. 25451426 10.1016/j.jad.2014.10.016

[24] Sanjuan J, Martin-Santos R, Garcia-Esteve L, Carot JM, Guillamat R, Gutierrez-Zotes A, et al. Mood changes after delivery: role of the serotonin transporter gene. Br J Psychiatry J Ment Sci. 2008;193:383–8. 10.1192/bjp.bp.107.045427 18978318

[25] Doornbos B, Dijck-Brouwer DAJ, Kema IP, Tanke MAC, van Goor SA, Muskiet FAJ, et al. The development of peripartum depressive symptoms is associated with gene polymorphisms of MAOA, 5-HTT and COMT. Prog Neuro-Psychopharmacol Biol Psychiatry. 2009;33:1250–4. 10.1016/j.pnpbp.2009.07.013 19625011

[26] Binder EB, Newport DJ, Zach EB, Smith AK, Deveau TC, Altshuler LL, et al. A serotonin transporter gene polymorphism predicts peripartum depressive symptoms in an at-risk psychiatric cohort. J Psychiatr Res. 2010;44:640–6. 20045118 10.1016/j.jpsychires.2009.12.001 PMC2891911

[27] Fan T, Hu Y, Xin J, Zhao M, Wang J. Analyzing the genes and pathways related to major depressive disorder via a systems biology approach. Brain Behav. 2020;10:e01502. 31875662 10.1002/brb3.1502 PMC7010578

[28] eQTLGen, 23andMe, the Major Depressive Disorder Working Group of the Psychiatric Genomics Consortium, Wray NR, Ripke S, Mattheisen M, et al. Genome-wide association analyses identify 44 risk variants and refine the genetic architecture of major depression. Nat Genet. 2018;50:668–81. 29700475 10.1038/s41588-018-0090-3 PMC5934326

[29] Howard DM, Adams MJ, Clarke T-K, Hafferty JD, Gibson J, Shirali M, et al. Genome-wide meta-analysis of depression identifies 102 independent variants and highlights the importance of the prefrontal brain regions. Nat Neurosci. 2019;22:343–52. 30718901 10.1038/s41593-018-0326-7 PMC6522363

[30] Kiewa J, Meltzer‐Brody S, Milgrom J, Guintivano J, Hickie IB, Whiteman DC, et al. Perinatal depression is associated with a higher polygenic risk for major depressive disorder than non-perinatal depression. Depress Anxiety. 2022;da.23232. doi:10.1002/da.23232. 34985809

[31] Guintivano J, Byrne EM, Kiewa J, Yao S, Bauer AE, Aberg KA, et al. Meta-analyses of genome-wide association studies for postpartum depression. Am J Psychiatry. 2023;180:884–95. 37849304 10.1176/appi.ajp.20230053 PMC11163373

[32] Ikeda M, Saito T, Kanazawa T, Iwata N. Polygenic risk score as clinical utility in psychiatry: a clinical viewpoint. J Hum Genet. 2021;66:53–60. 32770057 10.1038/s10038-020-0814-y

[33] Byrne EM, Carrillo-Roa T, Penninx BWJH, Sallis HM, Viktorin A, Chapman B, et al. Applying polygenic risk scores to postpartum depression. Arch Womens Ment Health. 2014;17:519–28. 25037970 10.1007/s00737-014-0428-5 PMC4341990

[34] Bauer AE, Liu X, Byrne EM, Sullivan PF, Wray NR, Agerbo E, et al. Genetic risk scores for major psychiatric disorders and the risk of postpartum psychiatric disorders. Transl Psychiatry. 2019;9:288. 31712652 10.1038/s41398-019-0629-9 PMC6848186

[35] Pouget JG, Taylor VH, Dennis C-L, Grigoriadis S, Oberlander T, Frey BN, et al. Preliminary insights into the genetic architecture of postpartum depressive symptom severity using polygenic risk scores. Pers Med Psychiatry. 2021;27–28:100081.

[36] Rantalainen V, Binder EB, Lahti-Pulkkinen M, Czamara D, Laivuori H, Villa PM, et al. Polygenic prediction of the risk of perinatal depressive symptoms. Depress Anxiety. 2020;37:862–75. 32627298 10.1002/da.23066

[37] Watanabe K, Taskesen E, van Bochoven A, Posthuma D. Functional mapping and annotation of genetic associations with FUMA. Nat Commun. 2017;8:1826. 29184056 10.1038/s41467-017-01261-5 PMC5705698

[38] Nurnberger JI, Blehar MC, Kaufmann CA, York-Cooler C, Simpson SG, Harkavy-Friedman J, et al. Diagnostic interview for genetic studies. Rationale, unique features, and training. NIMH Genetics Initiative. Arch Gen Psychiatry 1994;51:849–59; discussion 863–64. 7944874 10.1001/archpsyc.1994.03950110009002

[39] Turner S, Armstrong LL, Bradford Y, Carlson CS, Crawford DC, Crenshaw AT, et al. Quality control procedures for genome-wide association studies. Curr Protoc Hum Genet. 2011;68. 10.1002/0471142905.hg0119s68 PMC3066182 21234875

[40] Howie BN, Donnelly P, Marchini J. A flexible and accurate genotype imputation method for the next generation of genome-wide association studies. PLoS Genet. 2009;5:e1000529. 19543373 10.1371/journal.pgen.1000529 PMC2689936

[41] Dizier M-H, Demenais F, Mathieu F. Gain of power of the general regression model compared to Cochran-Armitage Trend tests: simulation study and application to bipolar disorder. BMC Genet. 2017;18:24. 28283021 10.1186/s12863-017-0486-6 PMC5345257

[42] Gloaguen E, Dizier M-H, Boissel M, Rocheleau G, Canouil M, Froguel P, et al. General regression model: A ‘model-free’ association test for quantitative traits allowing to test for the underlying genetic model. Ann Hum Genet. 2020;84:280–90. 31834638 10.1111/ahg.12372

[43] Chang CC, Chow CC, Tellier LC, Vattikuti S, Purcell SM, Lee JJ, et al. Second-generation PLINK: rising to the challenge of larger and richer datasets. GigaScience. 2015;4:7. 25722852 10.1186/s13742-015-0047-8 PMC4342193

[44] Machiela MJ, Chanock SJ. LDlink: a web-based application for exploring population-specific haplotype structure and linking correlated alleles of possible functional variants. Bioinforma Oxf Engl. 2015;31:3555–7. 10.1093/bioinformatics/btv402 PMC4626747 26139635

[45] de Leeuw CA, Mooij JM, Heskes T, Posthuma D. MAGMA: generalized gene-set analysis of GWAS data. PLoS Comput Biol. 2015;11:e1004219. 25885710 10.1371/journal.pcbi.1004219 PMC4401657

[46] GTEx Consortium. The GTEx Consortium atlas of genetic regulatory effects across human tissues. Science. 2020;369:1318–30. 32913098 10.1126/science.aaz1776 PMC7737656

[47] Kang HJ, Kawasawa YI, Cheng F, Zhu Y, Xu X, Li M, et al. Spatio-temporal transcriptome of the human brain. Nature. 2011;478:483–9. 22031440 10.1038/nature10523 PMC3566780

[48] Choi SW, O’Reilly PF. PRSice-2: Polygenic Risk Score software for biobank-scale data. GigaScience. 2019;8. 10.1093/gigascience/giz082 PMC6629542 31307061

[49] Stahl EA, Breen G, Forstner AJ, McQuillin A, Ripke S, Trubetskoy V, et al. Genome-wide association study identifies 30 loci associated with bipolar disorder. Nat Genet. 2019;51:793–803. 31043756 10.1038/s41588-019-0397-8 PMC6956732

[50] The 1000 Genomes Project Consortium. A global reference for human genetic variation. *Nature* . 2015:526:68–74. 10.1038/nature15393 PMC4750478 26432245

[51] Batt MM, Duffy KA, Novick AM, Metcalf CA, Epperson CN. Is postpartum depression different from depression occurring outside of the perinatal period? A review of the evidence. Focus Am Psychiatr Publ. 2020;18:106–19. 33162848 10.1176/appi.focus.20190045 PMC7587887

[52] Comasco E, Sylvén SM, Papadopoulos FC, Oreland L, Sundström-Poromaa I, Skalkidou A, et al. Postpartum depressive symptoms and the BDNF Val66Met functional polymorphism: effect of season of delivery. Arch Womens Ment Health. 2011;14:453–63. 21997575 10.1007/s00737-011-0239-x

[53] Figueira P, Malloy-Diniz L, Campos SB, Miranda DM, Romano-Silva MA, De Marco L, et al. An association study between the Val66Met polymorphism of the BDNF gene and postpartum depression. Arch Womens Ment Health. 2010;13:285–9. 20169377 10.1007/s00737-010-0146-6

[54] Alvim-Soares A, Miranda D, Campos SB, Figueira P, Romano-Silva MA, Correa H. Postpartum depression symptoms associated with Val158Met COMT polymorphism. Arch Womens Ment Health. 2013;16:339–40. 23636476 10.1007/s00737-013-0349-8

[55] Tan E-C, Lim H-W, Chua T-E, Tan H-S, Lee TM, Chen HY. Investigation of variants in estrogen receptor genes and perinatal depression. Neuropsychiatr Dis Treat. 2018;14:919–25. 29636617 10.2147/NDT.S160424 PMC5880413

[56] Schiller CE, Meltzer-Brody S, Rubinow DR. The role of reproductive hormones in postpartum depression. CNS Spectr. 2015;20:48–59. 25263255 10.1017/S1092852914000480 PMC4363269

[57] Edwards B, Galletly C, Semmler-Booth T, Dekker G. Antenatal psychosocial risk factors and depression among women living in socioeconomically disadvantaged suburbs in Adelaide, South Australia. Aust N Z J Psychiatry. 2008;42:45–50. 18058443 10.1080/00048670701732673

[58] Muglia P, Tozzi F, Galwey NW, Francks C, Upmanyu R, Kong XQ, et al. Genome-wide association study of recurrent major depressive disorder in two European case–control cohorts. Mol Psychiatry. 2010;15:589–601. 19107115 10.1038/mp.2008.131

[59] Bloomquist BT, Darlington DN, Mueller GP, Mains RE, Eipper BA. Regulated endocrine-specific protein-18: a short-lived novel glucocorticoid-regulated endocrine protein. Endocrinology. 1994;135:2714–22. 7988462 10.1210/endo.135.6.7988462

[60] Seth S, Lewis AJ, Galbally M. Perinatal maternal depression and cortisol function in pregnancy and the postpartum period: a systematic literature review. BMC Pregnancy Childbirth. 2016;16:124. 27245670 10.1186/s12884-016-0915-y PMC4886446

[61] Pizzagalli DA, Holmes AJ, Dillon DG, Goetz EL, Birk JL, Bogdan R, et al. Reduced Caudate and Nucleus Accumbens Response to Rewards in Unmedicated Subjects with Major Depressive Disorder. Am J Psychiatry. 2009;166:702–10. 19411368 10.1176/appi.ajp.2008.08081201 PMC2735451

[62] Van Cauwenberge MGA, Bouckaert F, Vansteelandt K, Adamson C, De Winter FL, Sienaert P, et al. A longitudinal study of the association between basal ganglia volumes and psychomotor symptoms in subjects with late life depression undergoing ECT. Transl Psychiatry. 2021;11:1–10. 33795659 10.1038/s41398-021-01314-w PMC8017007

[63] Sasaki A, Liotta AS, Luckey MM, Margioris AN, Suda T, Krieger DT, et al. Immunoreactive corticotropin-releasing factor is present in human maternal plasma during the third trimester of pregnancy. J Clin Endocrinol Metab. 1984;59:812–4. 6332823 10.1210/jcem-59-4-812

[64] Duan C, Cosgrove J, Deligiannidis KM. Understanding peripartum depression through neuroimaging: a review of structural and functional connectivity and molecular imaging research. Curr Psychiatry Rep. 2017;19:70. 28823105 10.1007/s11920-017-0824-4 PMC5617352

[65] Sacher J, Wilson AA, Houle S, Rusjan P, Hassan S, Bloomfield PM, et al. Elevated brain monoamine oxidase A binding in the early postpartum period. Arch Gen Psychiatry. 2010;67:468–74. 20439828 10.1001/archgenpsychiatry.2010.32

[66] Liang M, Yang JL, Bian MJ, Liu J, Hong XQ, Wang YC, et al. Requirement of regulated endocrine-specific protein-18 for development and expression of regulated endocrine-specific protein-18 isoform c in mice. Mol Biol Rep. 2011;38:2557–62. 21104147 10.1007/s11033-010-0394-6

[67] Figueroa JD, Garcia-Closas M, Humphreys M, Platte R, Hopper JL, Southey MC, et al. Associations of common variants at 1p11.2 and 14q24.1 (RAD51L1) with breast cancer risk and heterogeneity by tumor subtype: findings from the Breast Cancer Association Consortium. Hum Mol Genet. 2011;20:4693–706. 21852249 10.1093/hmg/ddr368 PMC3209823

[68] Zhi L, Yao S, Ma W, Zhang W, Chen H, Li M, et al. Polymorphisms of RAD51B are associated with rheumatoid arthritis and erosion in rheumatoid arthritis patients. Sci Rep. 2017;7:45876. 28361912 10.1038/srep45876 PMC5374468

[69] Mehta D, Rex-Haffner M, Sondergaard HB, Pinborg A, Binder EB, Frokjaer VG. Evidence for oestrogen sensitivity in perinatal depression: pharmacological sex hormone manipulation study. Br J Psychiatry. 2019;215:519–27. 30457060 10.1192/bjp.2018.234

[70] O’Brien S, Sethi A, Gudbrandsen M, Lennuyeux-Comnene L, Murphy DGM, Craig MC, et al. Is postnatal depression a distinct subtype of major depressive disorder? An exploratory study. Arch Womens Ment Health. 2021;24:329–33. 32666403 10.1007/s00737-020-01051-x PMC7979595

[71] Tebeka S, Le Strat Y, Dubertret C. Is parity status associated with bipolar disorder clinical features, severity or evolution? J Affect Disord. 2018;225:201–6. 28837954 10.1016/j.jad.2017.08.042

[72] Tebeka S, Godin O, Mazer N, Bellivier F, Courtet P, Etain B, et al. Clinical characteristics of bipolar disorders with postpartum depressive onset. Prog Neuro-Psychopharmacol Biol Psychiatry. 2020;107:110225. 10.1016/j.pnpbp.2020.110225 33347983

[73] Munk-Olsen T, Laursen TM, Meltzer-Brody S, Mortensen PB, Jones I. Psychiatric disorders with postpartum onset: possible early manifestations of bipolar affective disorders. Arch Gen Psychiatry. 2012;69:428–34. 22147807 10.1001/archgenpsychiatry.2011.157

[74] American Psychiatric Association. Diagnostic and Statistical Manual of Mental Disorders. 5th ed. DSM 5. 2013.

[75] Bodmer W, Bonilla C. Common and rare variants in multifactorial susceptibility to common diseases. Nat Genet. 2008;40:695–701. 18509313 10.1038/ng.f.136 PMC2527050

[76] Altshuler D, Daly M. Guilt beyond a reasonable doubt. Nat Genet. 2007;39:813–5. 17597768 10.1038/ng0707-813

