# Supplementary material for: Genome-wide association study of early-onset and late-onset postpartum depression: the IGEDEPP prospective study
Source: Eur Psychiatry. 2024 Apr 1;67(1):e35. doi: 10.1192/j.eurpsy.2024.26 (PMC11059250; doi:10.1192/j.eurpsy.2024.26)
Supplement: Tebeka et al. supplementary material [file S0924933824000269sup001.zip › 7.1 IGEDEPP_GWAS_Figure S2R.docx]

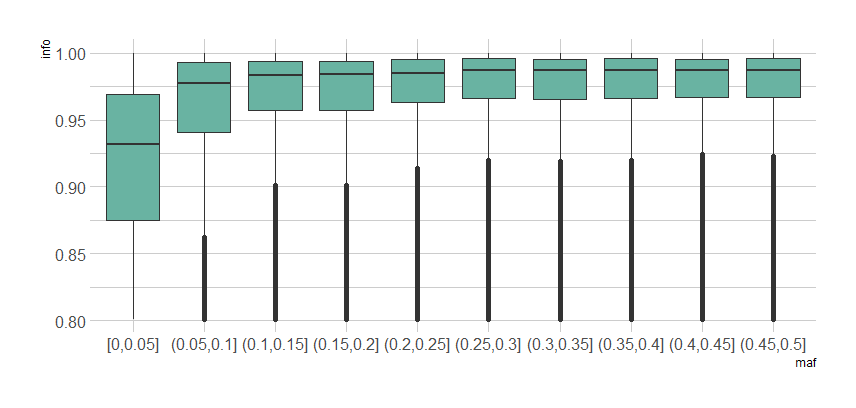


**Figure S2: Imputation quality (info score) of genetic variants with respect to their minor allele frequency (MAF)**
