# Supplementary material for: Genome-wide association study of early-onset and late-onset postpartum depression: the IGEDEPP prospective study
Source: Eur Psychiatry. 2024 Apr 1;67(1):e35. doi: 10.1192/j.eurpsy.2024.26 (PMC11059250; doi:10.1192/j.eurpsy.2024.26)
Supplement: Tebeka et al. supplementary material [file S0924933824000269sup001.zip › 7.2 IGEDEPP_GWAS_Figure S3R.docx]

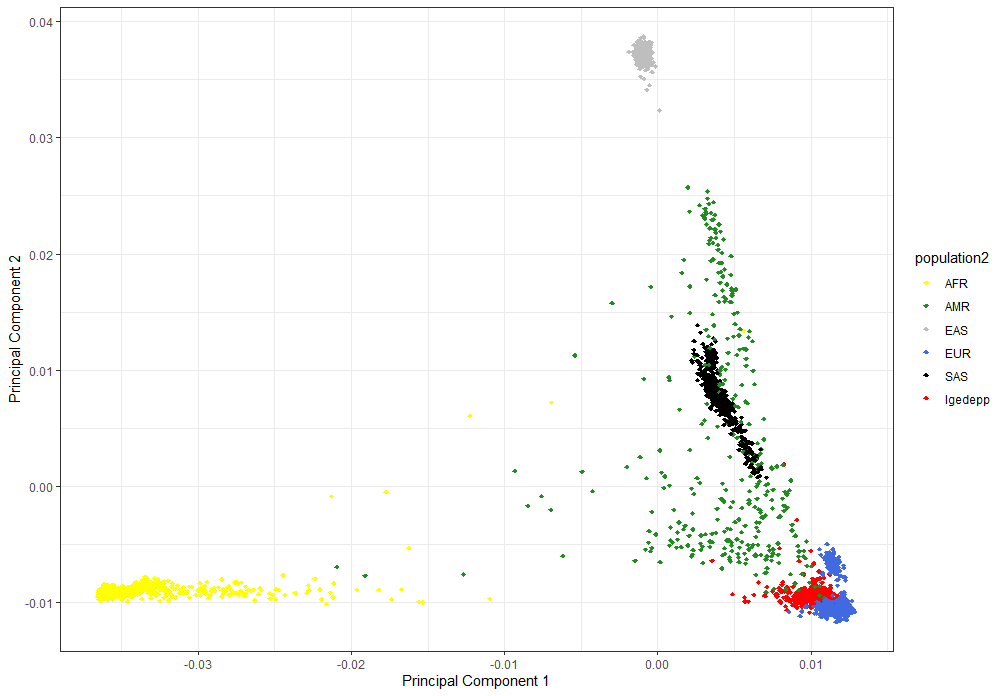


**Figure S3: PCA performed on IGEDEPP and 1000 Genomes Phase 3 superpopulations.**

PCA comparison to 1000 Genomes project population and IGEDEPP. Each color correspond to a population (AFR: African; AMR: Admixed American; EAS: East Asian; EUR: European; SAS: South Asian).
