# Supplementary material for: Genome-wide association study of early-onset and late-onset postpartum depression: the IGEDEPP prospective study
Source: Eur Psychiatry. 2024 Apr 1;67(1):e35. doi: 10.1192/j.eurpsy.2024.26 (PMC11059250; doi:10.1192/j.eurpsy.2024.26)
Supplement: Tebeka et al. supplementary material [file S0924933824000269sup001.zip › 7.7 IGEDEPP_GWAS_Figure S9R.docx]

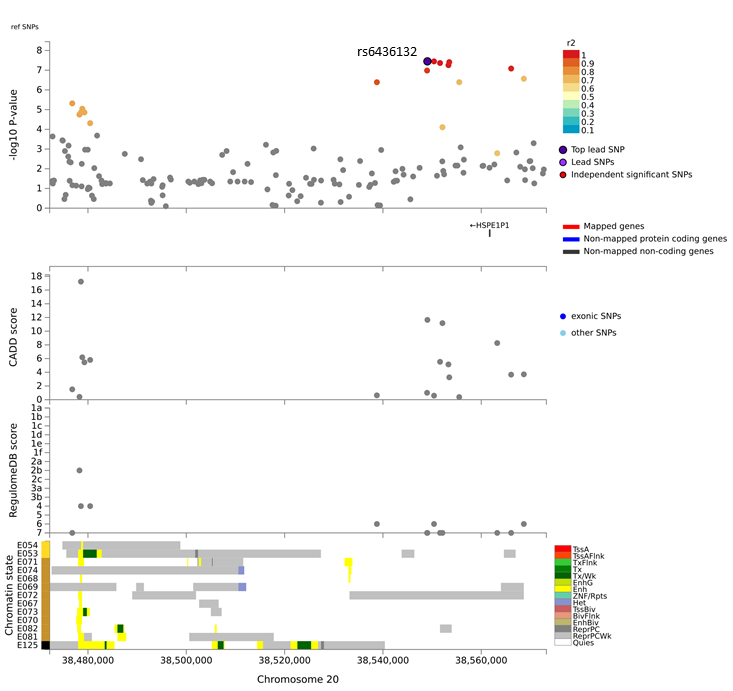


**Figure S9: Regional plot of locus 20q12 associated with late PPD.**

Regional plot for locus 20q12 with, from the top, GWAS P-value (SNPs are colored based on r²), CADD score, RequlomeDB score, chromatin state in the brain, eQTL P-value, Psych ENCORE promoter and Regulatory elements. Non-GWAS-tagged SNPs are shown at the top of the plot as rectangles since they do not have a P-value from the GWAS, but they are in LD with the lead SNP. eQTLs are plotted per gene and colored based on tissue types. In the plots of CADD score, RegulomeDB score and eQTLs.
