# Supplementary material for: Genome-wide association study of early-onset and late-onset postpartum depression: the IGEDEPP prospective study
Source: Eur Psychiatry. 2024 Apr 1;67(1):e35. doi: 10.1192/j.eurpsy.2024.26 (PMC11059250; doi:10.1192/j.eurpsy.2024.26)
Supplement: Tebeka et al. supplementary material [file S0924933824000269sup001.zip › 7.8 IGEDEPP_GWAS_Figure S10R.docx]

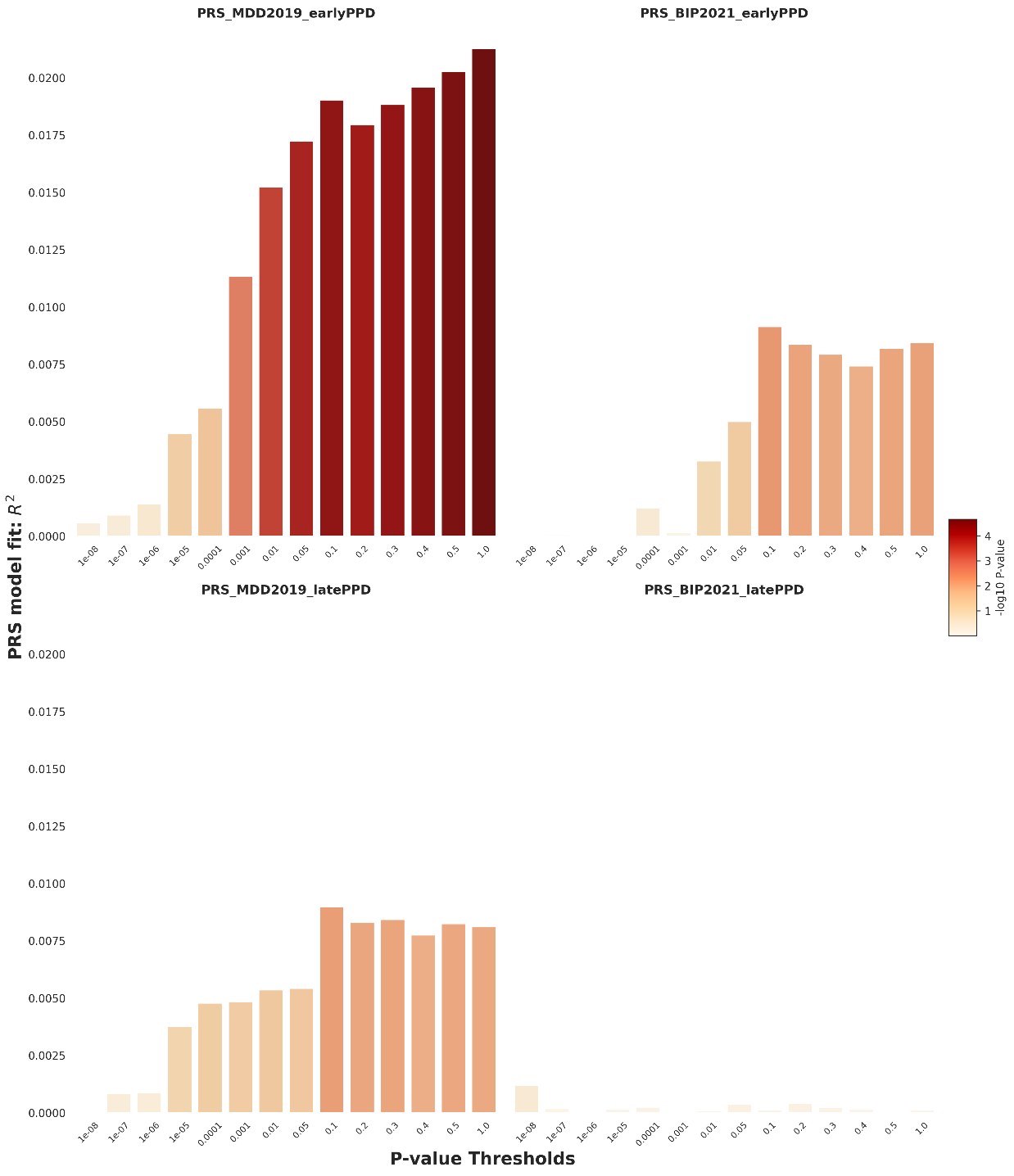


**Figure S10: General linear regression of PPD status on PRS with 14 different GWAS P-value thresholds**

upper left: PRS based on MDD2019 for early PPD, upper right: PRS based on BIP2021 for early PPD

lower left: PRS based on MDD2019 for late PPD, lower right: PRS based on BIP2021 for late PPD
